# Metabolic rates of different demographics in the sand fiddler crab *Leptuca pugilator*

**DOI:** 10.1371/journal.pone.0308617

**Published:** 2024-10-28

**Authors:** Carter Stancil, Nanette Smith, Laura S. Fletcher, Lars Anderson, Blaine D. Griffen

**Affiliations:** Department of Biology, Brigham Young University, Provo, Utah, United States of America; Universiti Malaysia Terengganu, MALAYSIA

## Abstract

Studies on animal energetics often focus on standardized metabolic rates to facilitate comparison across systems. Yet these standardized measurements often do not capture the realistic demographic and environmental variation that is common in natural settings. Rather, individuals included in these studies are often non-reproducing, uninjured, resting adults that have not recently eaten–far from a representative sample. We measured the respiratory rates of the sand fiddler crab *Leptuca pugilator* in air immediately after capture in the field, and compared rates between males, females of different reproductive states, and juveniles. As expected, we show that metabolic rates were influenced by body mass and activity level. We also show that being vitellogenic or gravid had only minor impacts on metabolic costs of females. Importantly, we demonstrate how considering demographics allows for the detection of phenomena that would otherwise go unnoticed. We found that field metabolic rates of *L*. *pugilator* in air are as much as an order of magnitude higher than previous standard metabolic rates measured on post-prandial, quiescent individuals. These higher rates may reflect a combination of high activity and active digestion, as fiddler crabs actively feed during low tide periods. Our results highlight the importance of considering differences in sex, life history stage, and reproductive state of organisms in fluctuating environments, such as intertidal habitats, when assessing energy expenditure.

## Introduction

The rate at which an organism uses energy provides insight into the connection between energy intake via consumption and the expenditure of energy to fuel essential biological processes. Because consumers influence energy flow by recirculating matter through ecosystems, understanding their metabolic rates is crucial to explaining ecosystem function [1]. An organism’s growth and reproduction are constrained by its energy budget; the amount of energy available for egg production depends on the amount consumed and the amount allocated to metabolic functions. Energy budgets and the study of energetics in general are valuable tools that have been used across a wide range of ecological settings and questions, to determine growth and reproductive costs [2], to understand breeding ecology and nesting [3], to explain maternal investment and sex allocation [4], and to predict physiological ecology and life histories [5]. The energetics of individual organisms are not constant, but vary with the dynamics of the ecosystems in which they live, as well as with their own individual state. The impacts of dynamic habitats are particularly apparent in ecosystems, such as marine intertidal habitats, that experience drastic daily shifts in physical conditions with the rising and falling of the tide.

Several general conceptual arguments suggest that an organism’s metabolic rate may differ in and out of water. For instance, during aerial exposure intertidal organisms experience broader temperature fluctuations, which greatly impacts metabolic rates of poikilotherms that predominate in intertidal habitats [6]. Additionally, intertidal organisms often display behaviors that correlate with tidal phase, such as increased foraging activity during high tide compared to low tide [7], that can strongly influence metabolic costs [8, 9]. These tidally-dependent feeding patterns not only influence metabolic costs by changing activity levels, but also alter temporal patterns in metabolic rates due to energetic costs of digestion (i.e., specific dynamic action) [10]. For intertidal species that are most active during low tide, assessing metabolic needs during low tide periods may provide a very different picture of energetics than in-water measurements that are more common in the literature.

Metabolic rate can also vary with several factors that are specific to the individual organism, such as body size [11, 12], activity level [13], injury [14], sex [15], or body condition [16]. An individual’s developmental stage and sex can also be expected to affect metabolic rate. In many species, juveniles allocate a large amount of their energy toward growth, to decrease the likelihood of being predated upon, to reach reproductive maturity more quickly, or to be able to better tolerate harsh environmental conditions [12, 17–19]. Once mature, their energy is divided between reproduction, continued growth (for some), maintenance, and other activities based on species-specific life history [20]. Differences in energy allocation with life history stage may also affect an organism’s contribution to overall ecosystem processes, further shaping their community. For instance, fish that grow to smaller body size experience higher individual turnover due to increased predation risk, and are thus responsible for a large amount of energy flux, relative to larger-bodied species, within coral reef communities [21].

Salt marsh systems are intertidal habitats that cover extensive coastal area throughout much of the southeastern part of the United States. The proportion of time that regions of the marsh are inundated vary widely with marsh elevation, ranging from <1% annually (in upper marsh regions) to >70% annually (in lower marsh regions) [22]. Previous work on salt marsh inhabitants suggests a significant difference between aerobic and aquatic energetics [23]. However, the energetic differences between demographic segments of populations has received little attention. Instead, energetics in marsh systems have often focused on populations through measuring energy use by ‘average’ individuals and then scaling this up based on the number of individuals in the population (e.g., [24, 25]). While it is important to look at overall energetics of a population, ignoring demographic differences between individuals can lead to an incomplete picture at the population level, and certainly fails to capture broadly representative energy dynamics at the individual level.

Fiddler crabs are intertidal decapods commonly found in salt marsh habitats around the world. The numerically abundant decapod in the salt marshes of southeastern North America is the sand fiddler crab, *Leptuca pugilator* (Bosc 1802; Crustacea, Ocypodidae; previously *Uca pugilator*). The geographic range of *L*. *pugilator* spans from Alabama to Florida in the Gulf of Mexico, and from Florida to Massachusetts on the Atlantic coast [26]. As with other species, demographic information such as sex and size have been consistently overlooked in examining the energetics of this species [23, 27–29]. Populations of this species are most abundant in upper marsh habitats where they spend the majority of their time exposed to aerial, low tide conditions. We used an in-situ closed system respirometry method [30] to measure aerial respiration rates of male and female *L*. *pugilator* of a range of sizes. Our measurements allowed us to examine how metabolic rate varied with temperature, body size, sex, reproductive state (vitellogenic and gravid), developmental stage, activity level, and limb loss and regrowth. We test the hypothesis that each of these factors influences the in-air energy expenditure of *L*. *pugilator* during low tide periods.

## Methods

### Preparation and collection

**Fig 1:**
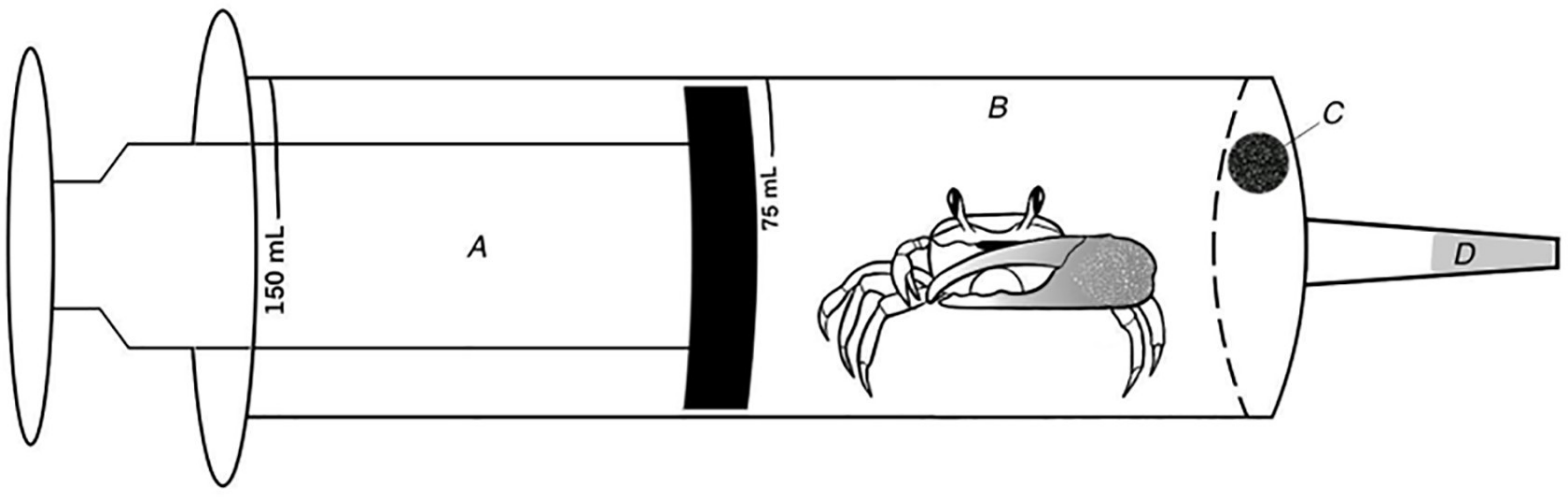
Diagram of experimental apparatus (150 ml syringe) used for measuring oxygen consumption. A) Plunger, B) barrel with crab inside, C) port that was covered by septa during trial to create a sealed chamber and through which the gas sample was extracted at the end of a trial, D) tip of chamber sealed with silicone.

Between July 12 and July 15, 2021, we collected crabs each morning during low tides from salt marshes within Hobcaw Barony in Georgetown, South Carolina, USA (33°20’5.61"N 79°11’36.34"W). We collected 274 crabs by hand and from burrows using trowels (since gravid females do not occur on the marsh surface). Of the 274 crabs, 158 were female (57.7%), 89 were male (32.5%), and 27 were juveniles (9.9%). All crabs were immediately transported to a screened-in outdoor lab for the Baruch Institute for Marine and Coastal Sciences run by the University of South Carolina. Crabs were then maintained in containers with lids (i.e., in shade conditions) with approximately 1 cm of water to maintain moist conditions while they waited to be measured. Crabs were measured in blocks of 10 at a time, and processing all individuals took an average of 4.5 hours each day. Following methods of Fletcher et al. [31], each was placed in a separate metabolic chamber made from clear 150-mL polypropylene syringes (length 14.9 cm, diameter 4.4 cm), the tips of which had been plugged with silicone. Each chamber also had an 8-mm diameter hole drilled into the end that served as a port for extraction of air samples (see Fig 1).

Depending on the size of the crab, we adjusted the chamber volume of the syringe, and thus the volume of air. The volume used was determined in preliminary trials and ranged from 30 ml for the smallest crabs to 120 ml for the largest crabs. This approach ensured that volumes were sufficiently small to ensure measurable changes during a trial, but sufficiently large to prevent hypoxic conditions due to oxygen depletion. Crabs were then left undisturbed for 5 minutes to adjust to the experimental chambers. While we made efforts not to disturb crabs during this adjustment period and subsequent measurement periods, handling has been shown in other crab species to elevate metabolic rates for several hours [32]. The extent of similar impacts in *L*. *pugilator* are not known. However, given that all crabs in our study were treated in an identical manner, these disturbance effects do not compromise our ability to make comparisons between crabs within our study. During this adjustment period, we measured the air temperature, relative humidity, and barometric pressure using a BTMeter (model 100-AAP).

### Trial procedure

Trial procedures followed those described by Fletcher et al. [31]. A trial was initiated by placing a septum (Bridge Analyzers, Inc.), designed for use in headspace analysis, over the sampling port and recording the time the chamber was sealed. The septa prevented any gas exchange between the chamber and environment, and also provided a surface that could be pierced by the gas probe’s needle at the end of the trials.

Throughout the duration of a trial, we observed the activity of each crab using visual scans at one-minute intervals. A crab was considered active if it was moving at all within the experimental chamber, and all movements were considered to be equal. During each scan, we recorded whether or not a crab was active. At the end of the trial, we summed the number of active observations to create an activity score and then used the trial duration to calculate the proportion of time active.

We varied the duration of the trial for individual crabs (20–90 minutes, longer for smaller crabs) so that, in combination with chamber volume, changes in oxygen concentration were detectable, but sufficiently minimal to avoid hypoxic conditions. The use of different chamber volume and trial duration for each crab did not affect our estimate of oxygen consumption, since these were explicitly included in the calculation of metabolic rate for each crab, as described below. Oxygen levels in experimental chambers always remained well above levels that cause anoxic conditions for air-breathing crustaceans [33]. Specifically, the minimum final O_2_ level encountered was 18.95%, with mean final levels at 20.04% ± 0.30%. Thus, the decrease was small compared to saturated atmospheric levels of 20.94%. Similarly, CO_2_ levels in experimental chambers always remained well below levels that cause hypercapnia [34]. Specifically, the maximum final CO_2_ level encountered was 0.99%, with mean final levels at 0.41% ± 0.16% (i.e., atmospheric levels 0.04%).

Each crab was used only once in this study. At the completion of a trial, we noted the time (for trial duration) and measured the final oxygen content of the chamber by inserting a needle through the port and extracting a gas sample using a multi-gas oxygen probe (Forensics Detectors^™^, model # FD-600, 0.01% resolution) with built-in pump (0.5 L/min). Crabs were then placed into individually-numbered sample bags and immediately frozen. Crabs were shipped on dry ice to Brigham Young University in Provo, UT and stored at -80°C until dissection.

### Dissection

We thawed the crabs to room temperature by submerging in room-temperature water. We then measured the carapace width, counted the number of missing limbs, and the number that were regenerating based on the presence of limb buds. Female crabs were inspected for evidence of eggs and were recorded as gravid or non-gravid. Female crabs were additionally dissected to determine whether they were vitellogenic. We did this by removing the ovary using dorsal dissection and examining it under a dissecting microscope to determine the presence of eggs. Crabs were determined to be “juveniles” if they were lacking external reproductive morphology and internal gonad tissue during dissection. To ensure sufficient sample size for analyses of juveniles, male and female juveniles were pooled together. We then dried crabs individually to constant weight at 60°C and the final dry weight was measured using a Mettler Toledo scale (DualRange model XS205).

### Calculations

We calculated metabolic rate for each crab individually using Equation 4.4 from [30]:

$$VolO_{2}=\frac{\left[V(F_{i}O_{2}\;-\;F_{e}O_{2})-\;F_{e}O_{2}(VolH_{2}O)\right]}{\left[1-F_{e}O_{2}(RQ)\right]},$$

where Vol*O*_*2*_ is the volume of oxygen consumed by the crab, *V* is the volume of the test chamber, *F*_*i*_*O*_*2*_ is the initial fractional oxygen concentration, *F*_*e*_*O*_*2*_ is the final fractional oxygen concentration, *VolH*_*2*_*O* is the volume of water vapor in the chamber, and *RQ* is the respiratory quotient.

To find *V*, the volume of air in the syringe chamber, we subtracted the crab’s volume from the recorded volume of the chamber. We determined crab volume by dividing the dry weight by 0.26, which is the empirically-determined factor used for crustaceans to convert dry weight to wet weight [35]. The volume is the wet weight divided by the density. Because crab density was an unknown variable, we used 1.1 g/cm^3^ for our equations (crabs sink in water, which has a density of 1.0 g/cm^3^), following Fletcher et al. [31]. These methods introduce uncertainty to our estimates of chamber volume; however, the degree of error remained low due to the small ratio of crab size to chamber volume.

*F*_*i*_*O*_*2*_ and *F*_*e*_*O*_*2*_, the initial fractional concentration of oxygen (i.e., 0.2094, the atmospheric concentration) and the fractional concentration at the end of the trial, were both measured at the time of the trials using the multi-gas oxygen probe as described above. We included a small amount of ambient temperature water (~0.5 ml) in each chamber during each trial so that water vapor was saturated [30]. We then determined the water vapor pressure using the relative humidity at the start of each trial and the mean temperature during each trial (measured at 10-min intervals throughout) and employing the water vapor pressure calculator found at respirometry.org. We used this estimate of water vapor pressure to calculate the Vol*H*_*2*_*0* as outlined in [30].

Finally, we set the respiratory quotient (RQ) to 0.85. Without knowing the exact ratio of protein, carbohydrates, and lipids in the diet, a value of 0.85 yields the lowest possible error from over- or underestimation of the RQ, with a maximum error of 3% [34]. However, our error was likely much lower than this because, as a deposit feeder, *L*. *pugilator* omnivorously consumes a combination of epipelic algae and meiofauna [35], and thus the assumed RQ of 0.85 is likely close to accurate.

After obtaining Vol*O*_*2*_, we used Equation 2.1 from [30] to correct the metabolic rates to standard temperature and pressure using the average temperatures (*T*) and barometric pressures (*BP*) that we measured during each trial. Finally, we divided standardized Vol*O*_*2*_ by the duration (minutes) of the trial for each crab and multiplied by 60 to convert oxygen consumption to a rate: volume oxygen consumed per hour.

### Statistical methods

Using R v.4.1.2, we used linear models to analyze data from male, female, and juvenile crabs separately. The response variable in each was the corrected mL O_2_ consumed per hour. Our predictor variables in the full model were dry mass (grams), proportion of time active during the trial, number of limbs missing, number of limbs regenerating, average ambient temperature during a trial (°C), as well as whether crabs were gravid or vitellogenic (for females). We first fit a full model that included all these predictor variables. We did not include any interaction terms because of the large number of predictor variables and because we had no *a priori* expectations about which interactions might be biologically or ecologically important. We then used the step function in the base package of R to determine the best-fitting model based on AIC [36].

In addition, for each demographic separately, we also used linear regression to determine whether there was a correlation between the ambient temperature and the time that crabs were active. The results of these analyses were used to further understand the results of the main analyses described above.

## Results

We measured oxygen consumption of 274 crabs total, including 158 adult females, 89 adult males, and 27 juveniles (sexes combined). Of these, 26 (16.5%) females were missing limbs (61 limbs missing total), with 21 of these missing limbs in the process of regenerating. Similarly, 7 (7.9%) males were missing limbs (36 limbs missing total), with 12 of these regenerating. Just 2 (7.4%) juveniles were missing limbs and 1 of these was regenerating. Most missing limbs were walking legs, with only 5 females missing a claw (3 regenerating) and 5 males missing a claw, 3 of which were major claws (4 regenerating). Of the females, 40 were gravid and 46 were vitellogenic. These were not mutually exclusive, as four females were both gravid and vitellogenic.

Overall, for males, females, and juveniles, oxygen consumption was influenced strongly by body size and was also influenced to varying degrees by activity level during the trials, by reproductive state, by injury, and by temperature during the trials. Below we describe these impacts for each of these groups individually.

**Fig 2:**
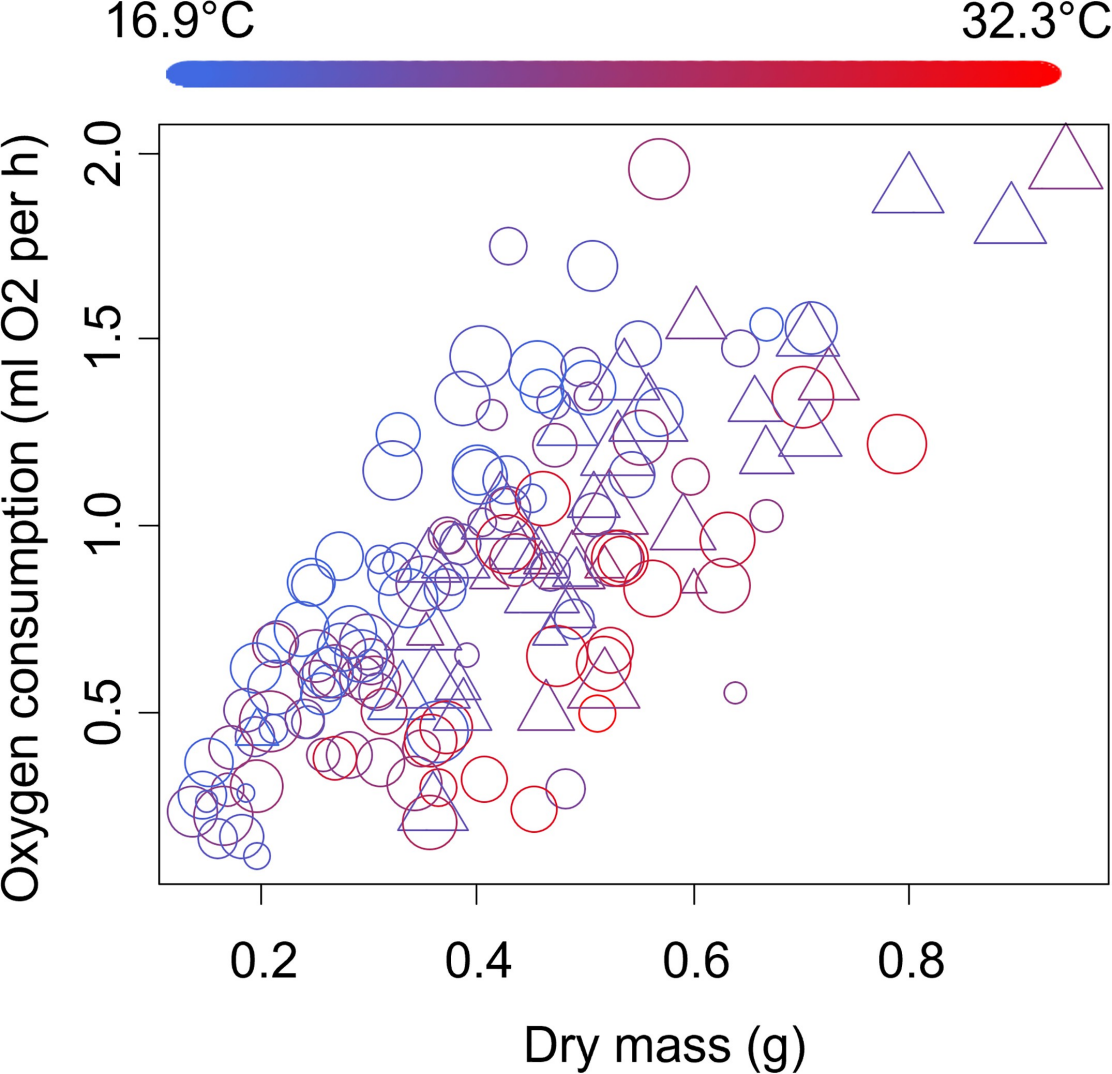
Oxygen consumption of adult male sand fiddler crabs, *Leptuca pugilator*. Symbols show number of limbs regenerating, where circle = no limbs regenerating; triangles = 1 limbs regenerating; plus = 2 limbs regenerating. Symbol size shows relative proportion of time active during the trial, where the smallest symbol is complete inactivity and the largest symbols are active throughout the trial. Symbol color shows the temperature during the trial, following the scale given on the top of the figure.

The oxygen consumption of male crabs increased by 1.41 ± 0.07 ml O_2_ per hour for every 1-gram increase in dry crab body mass (*t* = 20.26, *p* < 0.0001, Fig 2). The oxygen consumption of male crabs increased by 0.55 ± 0.13 ml O_2_ per hour as the proportion of time active during a trial increased (*t* = 4.35, *p* < 0.0001, Fig 2). Male crab oxygen consumption also increased by 0.15 ± 0.07 ml O_2_ per hour for each additional limb that was regenerating (*t* = 2.09, *p* = 0.04, Fig 2). The oxygen consumption of male crabs decreased slightly by -0.08 ± 0.01 ml O_2_ per hour for each 1°C increase in temperature (*t* = -10.59, *p* < 0.0001, Fig 2). No other variables were included in the best-fitting model.

**Fig 3:**
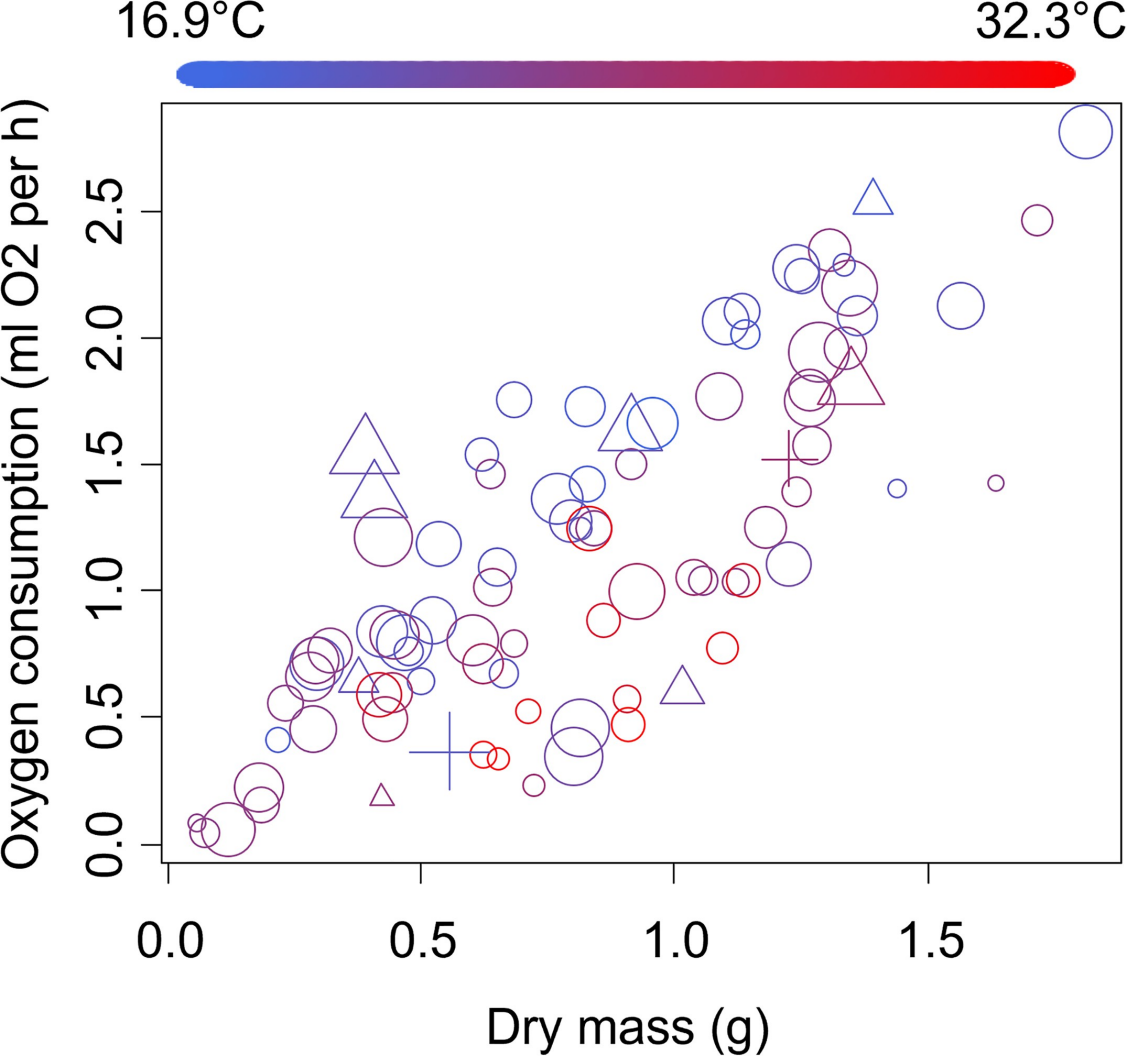
Oxygen consumption of adult female sand fiddler crabs, *Leptuca pugilator*. Circles show females that are not gravid, while triangles show females that are gravid. Symbol size and color are as described in the caption for Fig 2.

The oxygen consumption of female crabs increased by 2.23 ± 0.13 ml O_2_ per hour for every 1-gram increase in dry crab body mass (*t* = 16.77, *p* < 0.0001, Fig 3). Female crabs that were gravid had rates of oxygen consumption that were 0.15 ± 0.05 ml O_2_ per hour lower than females that were not gravid (*t* = -3.07, *p* = 0.003, Fig 3). Oxygen consumption also increased by 0.23 ± 0.10 ml O_2_ per hour as the proportion of time that crabs spent active increased (*t* = 2.36, *P* = 0.02, Fig 3), and decreased by 0.05 ± 0.01 ml O_2_ per hour for each 1°C increase in average temperature (*t* = -8.50, *P* < 0.0001). No other variables were included in the best-fitting model.

**Fig 4:**
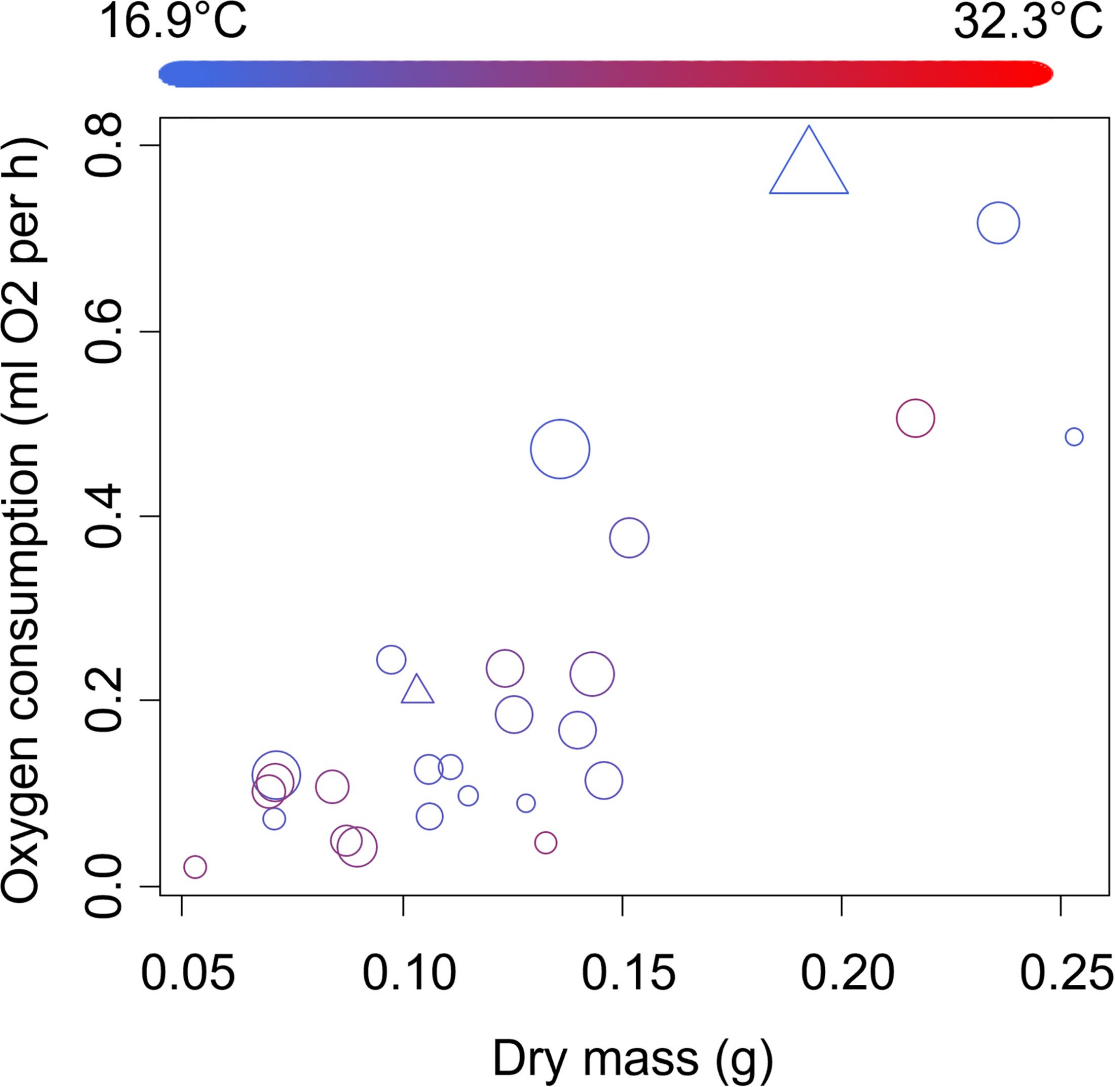
Oxygen consumption of juvenile sand fiddler crabs, *Leptuca pugilator*. Circles show crabs with no limbs missing, while triangles show crabs with a single limb missing. Symbol size and color are as described in the caption for Fig 2.

The oxygen consumption of juvenile crabs increased by 2.87 ± 0.33 ml O_2_ per hour for every 1-gram increase in dry crab body mass (*t* = 8.79, *p* < 0.0001, Fig 4). Oxygen consumption also increased by 0.31 ± 0.07 ml O_2_ per hour with an increase in the proportion of time that crabs were active during the trials (*t* = 4.19, *p* = <0.0003, Fig 4). No other variables were included in the best-fitting model.

We found that differences in activity level were weakly correlated (R^2^ = 0.02) with ambient temperature for adult females (*t* = 2.06, *P* = 0.04), while there was no change in activity level of adult males (*t* = -0.38, *P* = 0.7) or juveniles (*t* = -0.25, *P* = 0.81) with changes in average temperature.

## Discussion

As expected, the oxygen consumption rates for male, female, and juvenile *L*. *pugilator* were influenced by body mass and activity level. Though these differences should be expected, many studies of *L*. *pugilator* fail to include basic information on the size of study individuals. It follows that groups with various age, size, or sex ratios could expect to see differences in their overall oxygen consumption rates. We also found that oxygen consumption rates were influenced by the number of limbs missing (in juveniles only) and regenerating (in males only). Differences here were possibly due to vagaries driven by the relatively small numbers of individuals in each demographic that were missing or regenerating limbs. In female crabs, being gravid had a minor impact, while being vitellogenic had no impact. Counterintuitively, the oxygen consumption rate of adult males and females decreased with temperature, though this effect was small relative to other factors and was driven by a small number of data points where crabs were completely inactive (and therefore had low metabolism), but where ambient conditions were warm.

Our findings show that as the size of fiddler crabs increases, their oxygen consumption rates increase; however, the increase in oxygen consumption rates was much more dramatic with increases in body size for females and juveniles, compared to males. This suggests that males pay a lower cost for being large than that paid by females. The primary morphological difference between the sexes is the presence of a single enlarged claw in males. This strong sexual dimorphism in fiddler crabs has spurred previous studies to investigate the metabolic cost of the large male claw. Allen and Levinton [37] found that the presence of the claw increased metabolic costs for individuals by ~8% compared to declawed individuals. In contrast, previous work reported no significant differences in oxygen consumption between declawed and clawed male *L*. *pugilator* during rest or activity [38]. The actual metabolic cost of the single large claw in males is therefore not clear. Our findings suggest that the relatively low cost of greater body size in males compared to females may potentially compensate for any additional cost paid by the male associated with this enlarged claw.

In previous experiments [38], male *L*. *pugilator* had average resting in-air oxygen consumption rates of approximately 0.2–0.3 mL O2/g/hr [38]. A previous study examining the aerial oxygen consumption rate of *L*. *pugilator* found that oxygen consumption rates on land were 0.061 mL O_2_/g/hr [23]. Similar rates were found for male *L*. *pugilator* by Full and Herreid, who report a resting oxygen consumption rate of 0.049 ± 0.010 mL O2/g/hr [39]. However, oxygen consumption rates measured on all groups here were considerably higher than those reported in these previous studies. Several likely explanations explain this difference. First, these previous studies reported oxygen consumption per unit wet body mass, whereas we report rates per unit dry body mass. We did not measure wet body mass prior to drying animals and so cannot make direct comparisons. However, for decapods, dry body mass is on average 0.25 the wet body mass [35]. Thus, we would expect our rates to be 4-fold higher than these previously reported rates simply because of the use of dry vs. wet body masses.

Second, as mentioned above, oxygen consumption rates reported here were likely elevated due to stress from handling of experimental animals. Metabolism after handling can remain elevated in crabs for several hours [32], and thus surely had an impact on our study, though the magnitude of this impact is unknown. However, all individuals in our study were treated similarly, and these procedures should therefore not have influenced the relative oxygen consumption rates between demographic groups, or the impacts of reproduction and injury reported here. However, the absolute rates we report were almost certainly elevated by our handling procedures, likely explaining some of the difference between rates reported here and previously reported rates.

Third, our individuals were not in a resting state, but were active in the experimental chambers (65% of time on average, range 4–100%). Relatively few studies have measured metabolic scope in crabs, but a study on blue crabs (*Callinectes sapidus*) found that active oxygen consumption rates during vigorous exercise were approximately 5 times higher than resting rates [40]. A similar study on exercising ghost crabs (*Ocypode quadrata*) found oxygen consumption rates that were approximately 3.5 times higher than resting rates [39]. Assuming that the difference between active and resting rates are similar for *L*. *pugilator*, this likely explains a large amount of the high oxygen consumption that we measured. This is supported by the two previously mentioned studies of male *L*. *pugilator* that found oxygen consumption to increase up to 4.4 times [41] and 4–5 times [38] when crabs were exercised on a treadmill. Although our subjects were not being intentionally exercised, their increased activity, and therefore increased oxygen consumption, could perhaps be explained by the initial stress of collection and further handling.

Fourth, our crabs were captured in the field during the day when fiddler crabs are often actively feeding. Animals that have recently eaten have increased metabolism that represents the cost of digestion (i.e., specific dynamic action). Metabolism generally spikes for a few hours after eating, and then gradually declines as the remaining undigested food decreases. Studies in other crab species show that oxygen uptake spikes for about 5 hours after feeding, before it gradually declines again over the next 20 hours [10, 42], and that metabolism is about 3 times higher after food consumption compared to starved crabs [43]. *Post hoc* visual inspection of dissected dried crabs in our study revealed that 65% of them visibly had food in their cardiac stomach, which would have affected their oxygen consumption rates.

Fifth, the environmental temperature during our trials was highly variable, ranging from 16.9°C to 32.7°C. Increased temperatures have been shown to both increase and decrease the oxygen consumption rate of *Leptuca* species [29], and have also been linked to higher lactate dehydrogenase which may indicate increased energetic demand [44]. In our trials, temperature had a relatively modest effect by reducing oxygen consumption rates slightly in adult males and females. The reason for the decrease in metabolism with temperature is unclear, though similar results have been noted before in this species [29]. These authors hypothesized that metabolism may be depressed for individuals that are near their thermal maximum, which is 39.5°C [45]. However, this was unlikely the cause, as maximum temperatures during our metabolism trials was 32.7°C. It is also unclear how the temperatures we recorded relate to marsh surface temperatures experienced at our site by crabs during summer periods where crabs use behavioral thermoregulation via burrow use to modulate body temperatures [46].

Thus, several factors may be responsible for the relatively high absolute oxygen consumption rates we report here, and these rates may be more similar to ’field metabolic rates’ (i.e., metabolic rates expected of animals during normal day-to-day activity, digesting, etc.) as opposed to resting or standard metabolic rates when animals are inactive and not eating. Overall, while it is not clear how to interpret the absolute values we present, the relative values may be clearly compared across demographics given the similar methods used with all crabs in our study.

Our study fills several large knowledge gaps in our understanding of metabolism in *L*. *pugilator*. We looked at several nuanced situations that had not previously been examined in other studies, including maturity, reproductive state, missing limbs, regenerating limbs, and sex. According to our findings, being gravid did not increase the oxygen consumption rates of female crabs. Counterintuitively, our results showed a very slight decrease in the oxygen consumption rates of gravid females when compared to non-gravid individuals. This difference was statistically significant, but may have limited biological importance. These results suggest that for *L*. *pugilator*, carrying a clutch of eggs may not require large amounts of energy. However, it is possible that these low costs of egg carrying could reflect energy reallocation from other processes, such as growth and maintenance [47–49]. Gravid females were extracted from burrows where they generally remain while they are gravid [50], during which time they are presumably relatively inactive.

Interestingly, being vitellogenic did not appear to be metabolically expensive in *L*. *pugilator*. A possible explanation is that females are using lipids already stored in the hepatopancreas, rather than producing new lipids. Known patterns of lipid storage in other crab species are consistent with this explanation. Alava et al. [51] demonstrated in the mud crab *Scylla serrata*, that ovary lipid content peaked during late vitellogenesis and corresponded with a decline in hepatopancreas lipid content. It is possible that *L*. *pugilator* similarly mobilizes stored lipids from the hepatopancreas to produce their ova. In this case, the metabolic cost of vitellogenesis may have largely been paid previously during the process of lipid synthesis in the hepatopancreas. Further research is necessary to examine this hypothesis and to examine the cost of lipid mobilization in crabs.

Although previous work examines *L*. *pugilator* limb regrowth in different salinities and chemical stressors [52], and the effect of losing the major chela on heart rate [53], no study has previously looked at the metabolic cost of missing limbs, including walking legs. We found that adult male crabs experienced an increase in oxygen consumption rate with the number of limbs regenerating (juveniles had an increase in metabolism with the number of limbs missing, but this reflected just two individual animals and should not be overintepreted). This increased cost during limb regeneration may reflect the energy of protein synthesis for replacing lost limbs [54], and would likely be compounded with additional associated costs of limb loss, including increased energy requirements related to moving, feeding, mating, predator avoidance, etc. that were not measured in this study.

In summary, our results provide an important comparison between oxygen consumption rates of male, female, and juvenile *L*. *pugilator* under similar field conditions, and simultaneously examine other individual physiological states that may influence metabolism, including reproductive state and nonlethal injury. Our field methods can be easily adapted to measure low tide oxygen consumption rates in other marsh invertebrates in the field immediately following collection. Our results highlight the importance of considering individual variation when attempting to draw conclusions about energy expenditure of the larger population.

### Geolocation information

This study was conducted at the following location: Hobcaw Barony in Georgetown, South Carolina, USA (33°20’5.61"N 79°11’36.34"W).

## Supporting information

S1 Data This file contains all data used for the analysis in the current paper. (CSV)
